# Modulation of glycogen synthase kinase-3β following TRAIL combinatorial treatment in cancer cells

**DOI:** 10.18632/oncotarget.11834

**Published:** 2016-09-02

**Authors:** Sreevidya Santha, Gantulga Davaakhuu, Aninda Basu, Rong Ke, Subhasis Das, Ajay Rana, Basabi Rana

**Affiliations:** ^1^ Department of Surgery, Division of Surgical Oncology, University of Illinois at Chicago, Chicago, IL 60612, USA; ^2^ University of Illinois Hospital and Health Sciences System Cancer Center, University of Illinois at Chicago, Chicago, IL 60612, USA; ^3^ Jesse Brown VA Medical Center, Chicago, IL 60612, USA

**Keywords:** GSK3β, AKT, PPARγ, AMPK, apoptosis-resistance

## Abstract

Glycogen Synthase Kinase-3β (GSK3β) is a serine/threonine kinase, known to regulate various cellular processes including proliferation, differentiation, survival, apoptosis as well as TRAIL-resistance. Thus pathways that can modulate GSK3β axis are important targets for cancer drug development. Our earlier studies have shown that combinatorial treatment with Troglitazone (TZD) and TRAIL can induce apoptosis in TRAIL-resistant cancer cells. The current studies were undertaken to investigate whether GSK3β pathway was modulated during this apoptosis. Our results indicated an increase in inhibitory GSK3β^Ser9^ phosphorylation during apoptosis, mediated via AKT. At a later time, however, TZD alone and TRAIL-TZD combination produced a dramatic reduction of GSK3β expression, which was abolished by cycloheximide. Luciferase assays with GSK3β-luc promoter reporter showed that TZD can effectively antagonize GSK3β promoter activity. Since TZD is a ligand for transcription factor PPARγ and can activate AMPK, we determined their roles on antagonism of GSK3β. Knockdown of PPARγ was unable to restore GSK3β expression or antagonize GSK3β^Ser9^ phosphorylation. Although pretreatment with Compound C (pharmacological inhibitor of AMPK) partially rescued GSK3β expression, knockdown of AMPKα1 or α2 alone or in combination were ineffective. These studies suggested a novel PPARγ-AMPK-independent mechanism of targeting GSK3β by TZD, elucidation of which might provide newer insights to improve our understanding of TRAIL-resistance.

## INTRODUCTION

Glycogen synthase kinase-3 (GSK-3) is a multifunctional serine/threonine kinase that has been implicated in regulating several fundamental processes including cell proliferation, differentiation, metabolism, survival and apoptosis [1], as well as various pathological conditions such as diabetes, oncogenesis and neurological diseases [2]. GSK3 derived its name from its phosphorylation activity toward glycogen synthase, thus linking it to glycogen metabolism. Since then, increasing research on GSK3 has significantly improved our understanding of this molecule. Two GSK-3 genes (α and β) have been cloned in mammals with strong sequence conservation within the catalytic domain between the homologues [3]. Due to its profound role in neurodegeneration, the efficacy of GSK3 inhibitors in Alzheimer's disease have also been tested [4]. GSK3 is known to phosphorylate and regulate the activities of more than 40 proteins, many of which are transcription factors [5]. This indicates the potential contribution of this enzyme in regulating a variety of cellular functions. The regulation of GSK3β activity is not completely understood and is believed to be mediated via a combination of phosphorylation, localization and interaction with other proteins [6, 7]. The major inhibitory phosphorylation is on Ser9 of GSK3β and Ser21 of GSK3α [8, 9] and can be phosphorylated by multiple upstream kinases including AKT [10].

In the cancer field, GSK-3β is commonly recognized as a putative tumor suppressor due to its well-established function as a repressor of β-catenin signaling [11] and phosphorylation-dependent down-regulation of cell-cycle regulators cyclin D1 [12], cdc25 [13], and c-Myc [14]. Paradoxically, it can also promote cell survival and oppose apoptosis [15, 16]. An involvement of GSK3β in mediating tumorigenic pathways is also indicated by its induced expression in various cancers including colon cancer [17], pancreatic cancer [18, 19], prostate cancer [20–22], and glioblastoma [23]. This notion is supported by recent studies suggesting an involvement of GSK3β in pancreatic cancer cell survival [24], dedifferentiation [19] as well as therapeutic resistance [25–27]. Similarly, GSK3β inhibition was shown to ameliorate apoptosis resistance in other types of cancer as well [28, 29]. A close connection of GSK3β in prostate cancer has been demonstrated earlier by the fact that increased cytoplasmic GSK3β correlated with the clinical stage and Gleason score in prostate tumor samples [30]. In addition, GSK3β was shown to positively regulate androgen receptor (AR) function [31, 20] and nuclear translocation [32]. An understanding of how GSK3β pathway is modulated during apoptotic signaling is thus important for the development of new and effective therapeutic approaches that can target GSK3β.

In recent studies with TRAIL-resistant cancer cells, we have observed that treatment with a combination of TRAIL and PPARγ ligand Troglitazone (TZD) induces profound apoptosis compared to either agent alone [33]. The aim of the present study was to determine whether GSK3β pathway was modulated by this combination treatment and to elucidate the potential mechanism. Our results indicate that in TRAIL-resistant prostate cancer and hepatocellular carcinoma (HCC) cells, TRAIL and TZD treatment resulted in an induction of GSK3β^Ser9^ phosphorylation (indicating inhibition) at an earlier stage during apoptosis. In addition, total GSK3β levels were significantly down-regulated by TZD alone and by TRAIL-TZD combination at a later stage that involved inhibition of transcription. This downregulation of GSK3β involved mechanisms independent of PPARγ and AMPK. In addition, although pharmacological inhibition was ineffective, knockdown of GSK3α and to a lesser extent GSK3β seemed to promote apoptosis when treated with TRAIL-TZD combination.

## RESULTS

### Combination treatment with TRAIL-TZD attenuates GSK3β pathway in cancer cells

**Figure 1:**
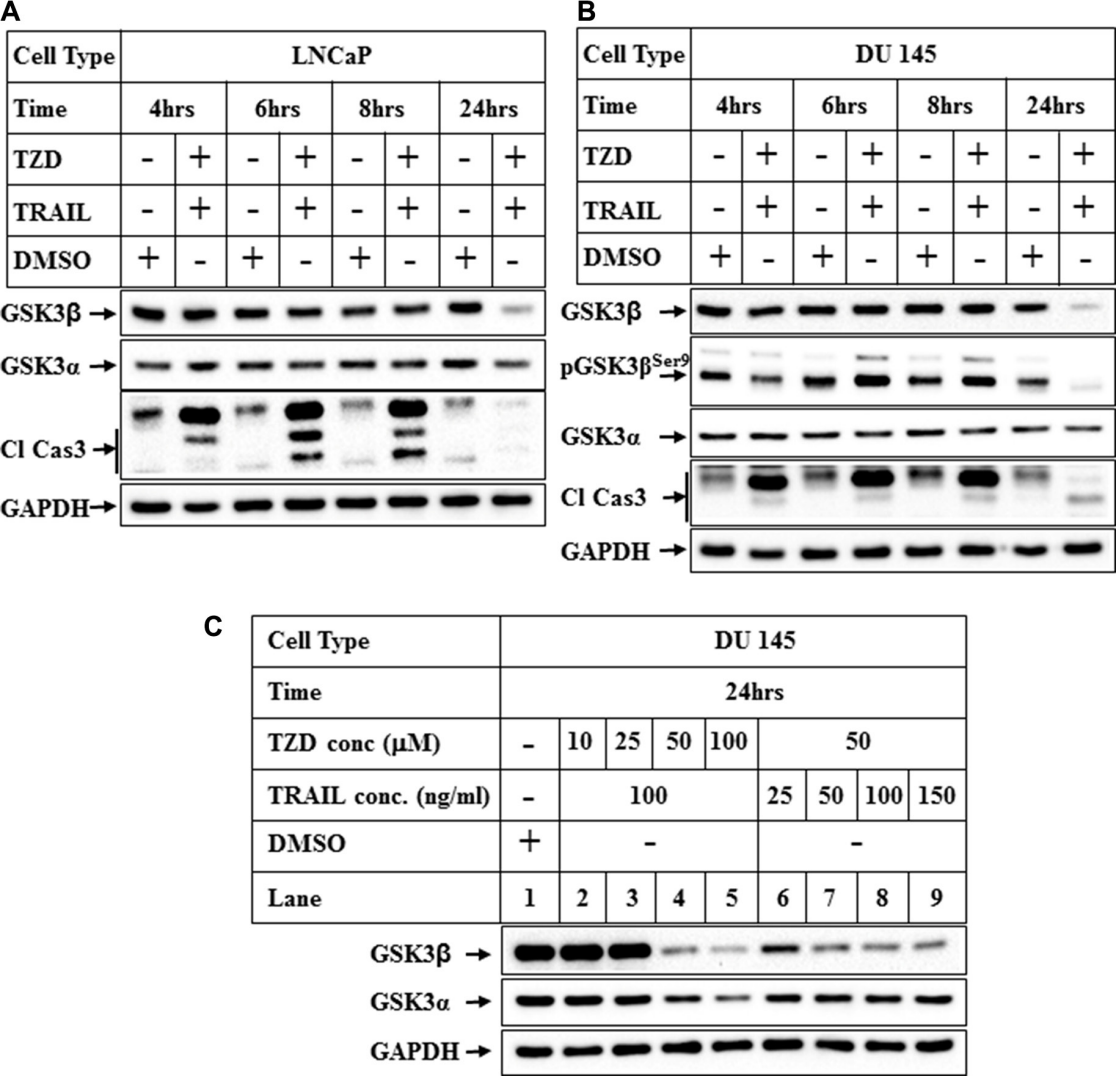
Attenuation of GSK3β pathway by combination treatment with TRAIL and TZD in prostate cancer cells (**A**) LNCaP and (**B**) DU 145 cells were treated with a combination of TRAIL (100 ng/ml) and Troglitazone (50 μM) for different periods of time followed by Western Blot analysis with the indicated antibodies. (**C**) DU 145 cells were treated with increasing concentrations of TZD (10, 25, 50, 100 μM), or with increasing concentrations of TRAIL (25, 50, 100, 150 ng/ml) for 24 hrs followed by Western Blot analysis with the indicated antibodies.

To understand the status of GSK3β pathway during apoptosis following treatment with TRAIL-TZD combination, prostate cancer cells (LNCaP and DU145) were treated with TRAIL-TZD combination for different lengths of time and changes in GSK3β levels were compared. Although total GSK3β expression was unaffected initially, prolonged treatment with TRAIL-TZD resulted in a significant reduction of total GSK3β expression in both cell types (Figure 1A, 1B 24 hrs). Reduction of total GSK3α, however was more modest. Similar regulation of GSK3β in both LNCaP (AR dependent) and DU145 (AR independent) cells suggested that this might be occurring independent of AR signaling. In addition, we also observed an increase of pGSK3β^Ser9^ levels, which preceded reduction of total GSK3β and coincided with the period of active apoptosis (Figure 1B). The concentrations of both TRAIL and TZD needed to inhibit total GSK3β expression optimally were determined next. These studies designed with increasing concentrations of each agent indicated that combination of 100 ng/ml TRAIL and 50–100 μM TZD produced maximal effects on total GSK3β expression (Figure 1C). Thus the remaining studies were carried out with this combination.

**Figure 2:**
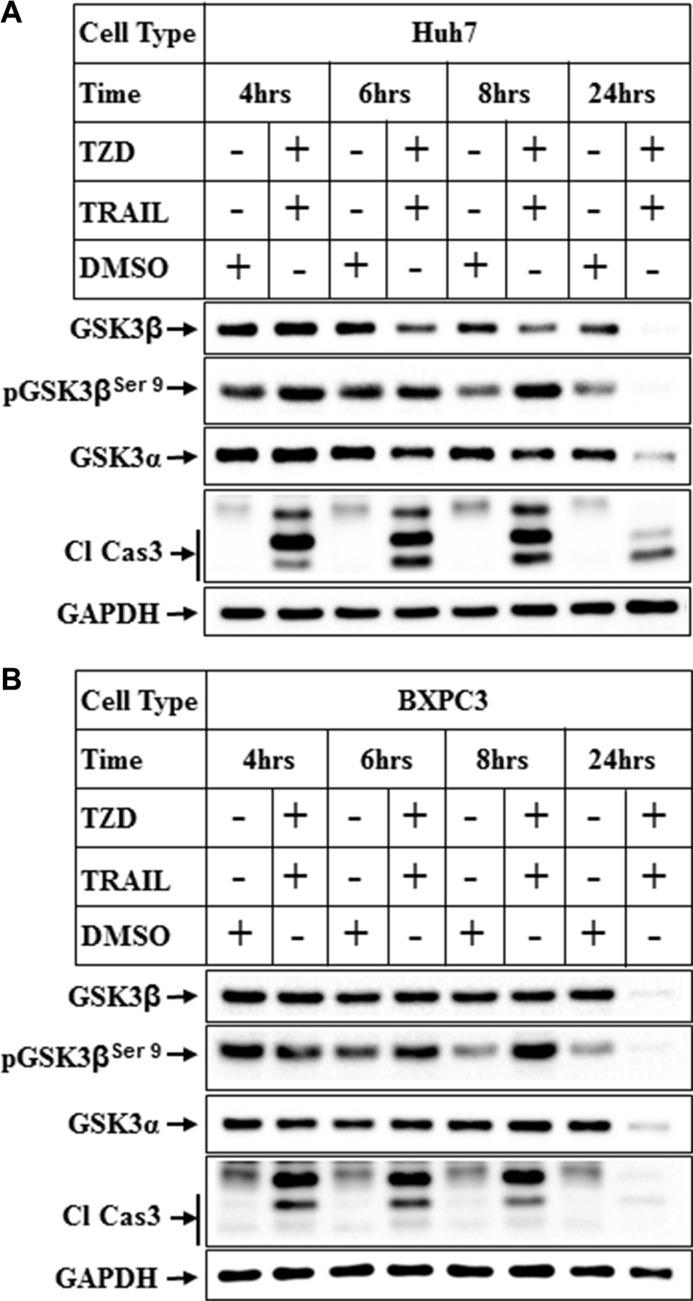
Attenuation of GSK3β pathway in various cancer cells by TRAIL and TZD treatment (**A**) Huh7 and (**B**) BxPC3 cells were treated with a combination of TRAIL (100 ng/ml) and Troglitazone (50 μM) for different periods of time followed by Western Blot analysis with the indicated antibodies.

In order to determine whether this regulation of GSK3β is also present in other cancer cells, we determined the changes in GSK3β levels in hepatocellular carcinoma cells (Huh7) and pancreatic cancer cells (BXPC3). These also showed a dramatic reduction of total GSK3β and α expressions in Huh7 cells following treatment with TRAIL-TZD combination (Figure 2A). A similar time dependent reduction of total GSK3β and α was also observed in the BXPC3 cells (Figure 2B). These were also associated with an initial increase in the levels of pGSK3β^Ser9^ in both cell types. These suggested that TRAIL-TZD-induced modulation of GSK3β pathway is present in various cancer cells and is a generalized event.

### Comparison of the effects of TRAIL and TZD alone or in combination in antagonizing GSK3β

**Figure 3:**
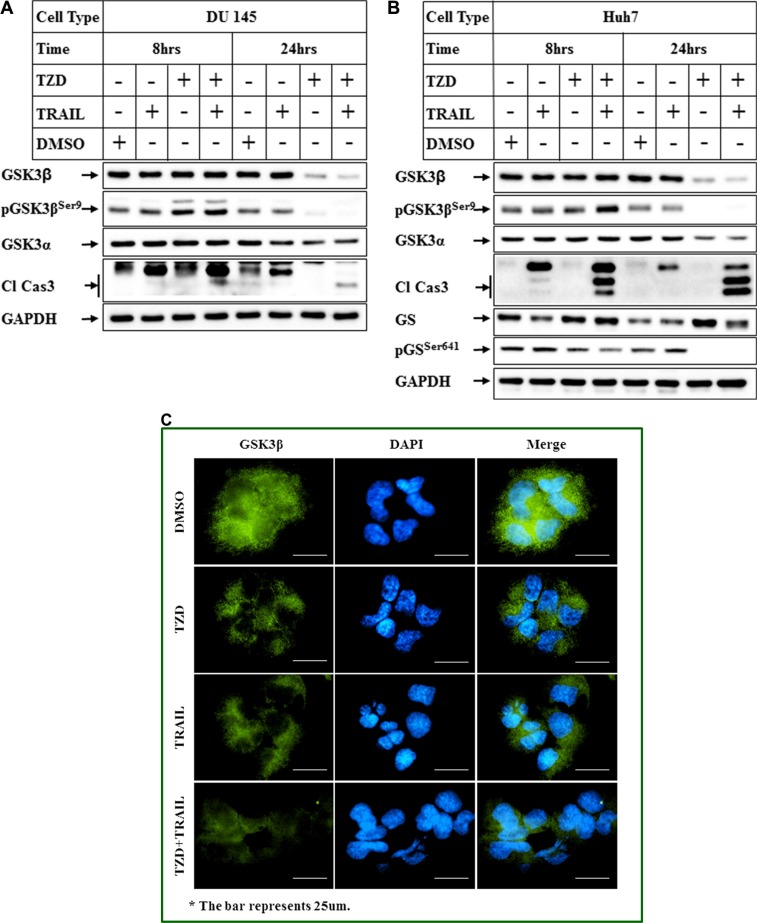
Comparison of the effects of TRAIL and TZD alone or in combination in antagonizing GSK3β pathway (**A**) DU 145 and (**B**) Huh7 cells were treated with DMSO, TRAIL or TZD or a combination of TRAIL and TZD for 8 hrs and 24 hrs. Western Blot analyses were performed next with the indicated antibodies. (**C**) Huh7 cells plated in 4-well chamber slides were treated with DMSO, TZD, or TRAIL alone or in combination for 8 hrs. Immunofluorescence analysis with anti-GSK3β antibody was performed to detect endogenous GSK3β (green fluorescence). The nuclei were stained with DAPI (blue). The images were captured on a Nikon ECLIPSE Ti microscope, equipped with a digital camera (Nikon DS-Qi2) at 40× magnification.

To understand the relative contribution of TRAIL or TZD alone and their combination in modulating total GSK3β expression, cells were treated with either agent alone or in combination and the effect on GSK3β levels was estimated. These studies indicated that treatment with TZD alone resulted in a significant attenuation of total GSK3β expression, which was further potentiated by TRAIL-TZD combination (Figure 3A, 3B). Antagonism of GSK3β axis by TZD and TRAIL-TZD was also evident from the reduction of GSK3β downstream target Glycogen Synthase Ser641 phosphorylation (Figure 3B, pGS^Ser641^ panel). To determine whether TRAIL-TZD induced any change in GSK3β localization, Immunofluorescence studies were designed. These showed that treatment with TZD alone or in combination with TRAIL significantly reduced cytoplasmic and nuclear GSK3β levels (Figure 3C). These suggested TZD to be a major modulator of GSK3β expression.

### TZD inhibits total GSK3β at a transcriptional level

**Figure 4:**
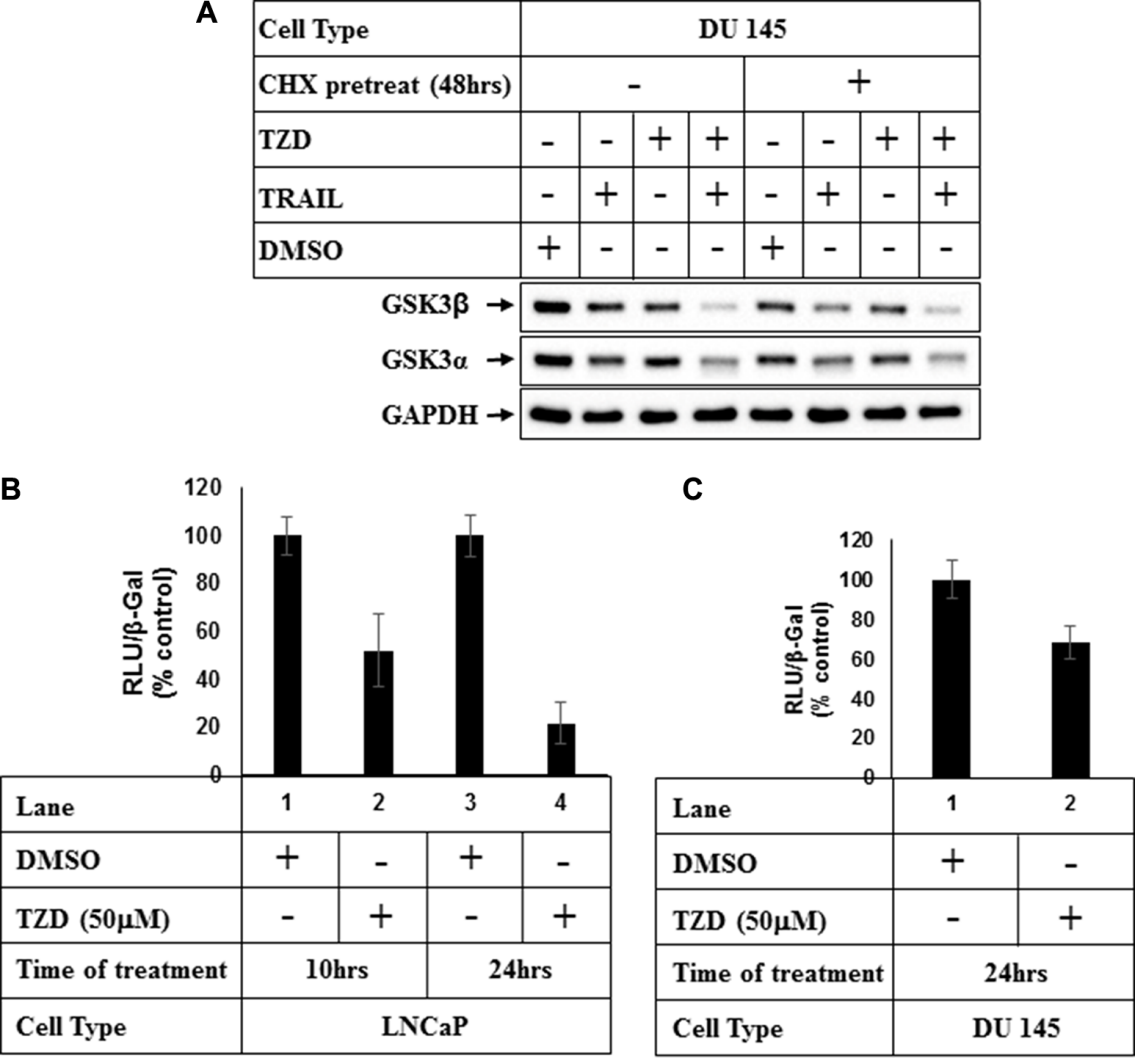
TZD treatment attenuates GSK3β expression at a transcriptional level (**A**) DU 145 cells were pretreated with 50 μg/ml Cycloheximide (CHX) for 48 hrs and then with DMSO or TRAIL, TZD alone or in combination for an additional 24 hrs. At the end of incubation, cells were harvested and equal amounts of total protein were analyzed by Western Blots with the antibodies indicated. (**B**) LNCaP or (**C**) DU 145 cells were transiently transfected with GSK-3β promoter luciferase vector [pGL3-GSK-3β-luc-(−427/+66)] and β-Galactosidase (β-Gal) vector for 48 hrs followed by treatment with DMSO, or TZD for the indicated periods of time. Luciferase and β-galactosidase assays were carried out next and RLU/β-Gal values were expressed as % control. The data in each set represent the mean ± SD of three independent experiments.

In an attempt to understand the mechanism how TZD regulates total GSK3β, studies were undertaken to determine whether the effects were at a post-translational level. Pretreatment with protein synthesis inhibitor cycloheximide (CHX) showed that although TZD reduced total GSK3β expression significantly in the absence of CHX (Figure 4A, compare lanes 1&3), it was unable to do so in the presence of CHX (compare lanes 5&7). These suggested that TZD attenuated total GSK3β levels not at a post-translational step and most likely regulated it at a transcriptional level or via modulating mRNA stability. To confirm any transcriptional regulation of GSK3β, we performed luciferase assays with GSK3β-promoter luciferase construct pGL3-GSK-3β-luc (−427/+66) [34] following treatment with TZD. These showed a significant attenuation of GSK3β promoter activity with TZD treatment in both LNCaP and DU145 cells in a time dependent manner (Figure 4B and 4C), suggesting that TZD can inhibit GSK3β transcription.

### TZD-induced attenuation of GSK-3β is PPARγ-independent

**Figure 5:**
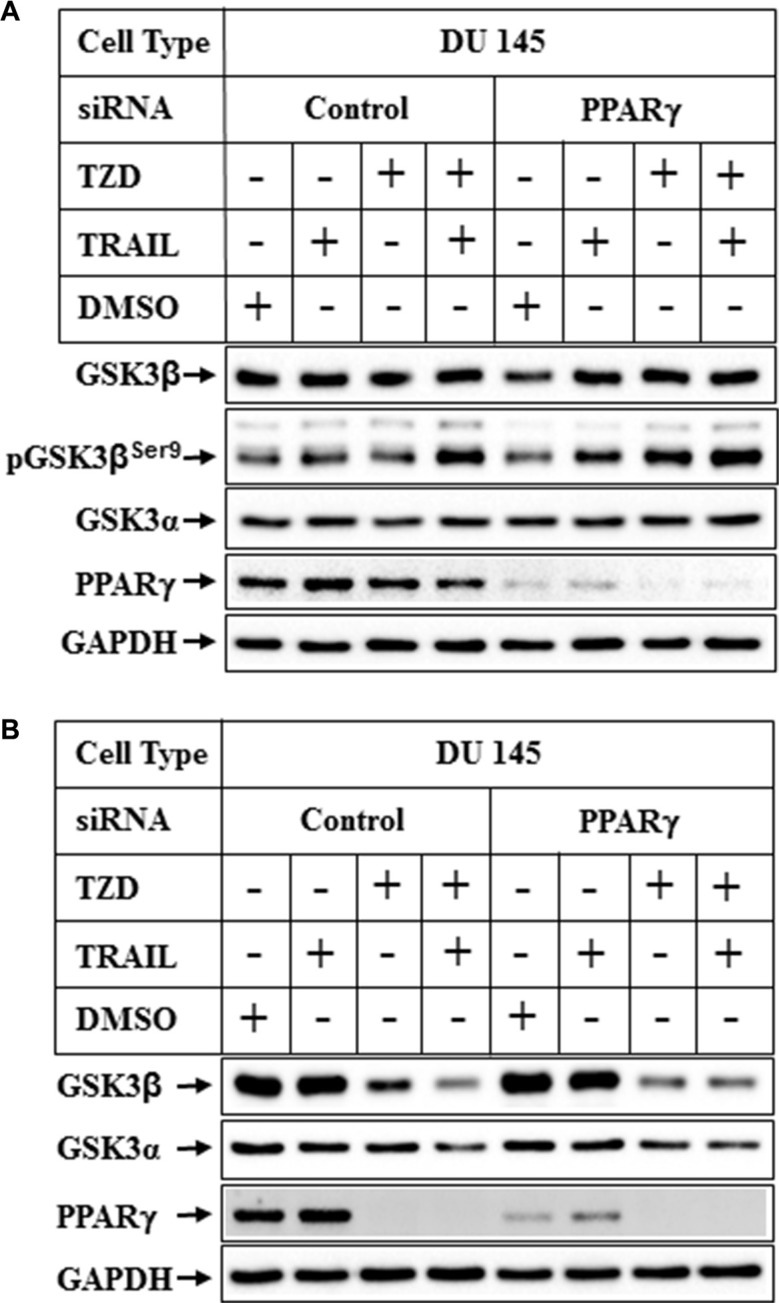
Effect of PPARγ knockdown on TRAIL-TZD-induced modulation of GSK3β pathway Subconfluent DU 145 cells were transfected with either control-siRNA or PPARγ-siRNA for 72 hrs followed by treatment with TRAIL or TZD alone or in combination for 6 hrs (**A**) or 24 hrs (**B**). The samples were analyzed by Western blots with the antibodies indicated.

Since TZD is a ligand for transcription factor PPARγ, and GSK3β was regulated at a transcriptional level by TZD, we determined next whether PPARγ played any role in modulating GSK3β expression. To understand the role of PPARγ on GSK3β expression, endogenous PPARγ expression was knocked down using the smart pool hPPARγ-siRNA from Dharmacon. PPARγ-siRNA reduced PPARγ expression significantly (Figure 5A, 5B, PPARγ-siRNA lanes), but was unable to antagonize TZD or TRAIL-TZD-induced reduction of total GSK3β expression (Figure 5B, compare lanes 3&4 with 7&8). These studies also revealed that, TRAIL-TZD-mediated induction of pGSK3β^Ser9^ was independent of PPARγ (Figure 5A).

### TZD-induced attenuation of GSK-3β is modulated by compound C independent of AMPK

**Figure 6:**
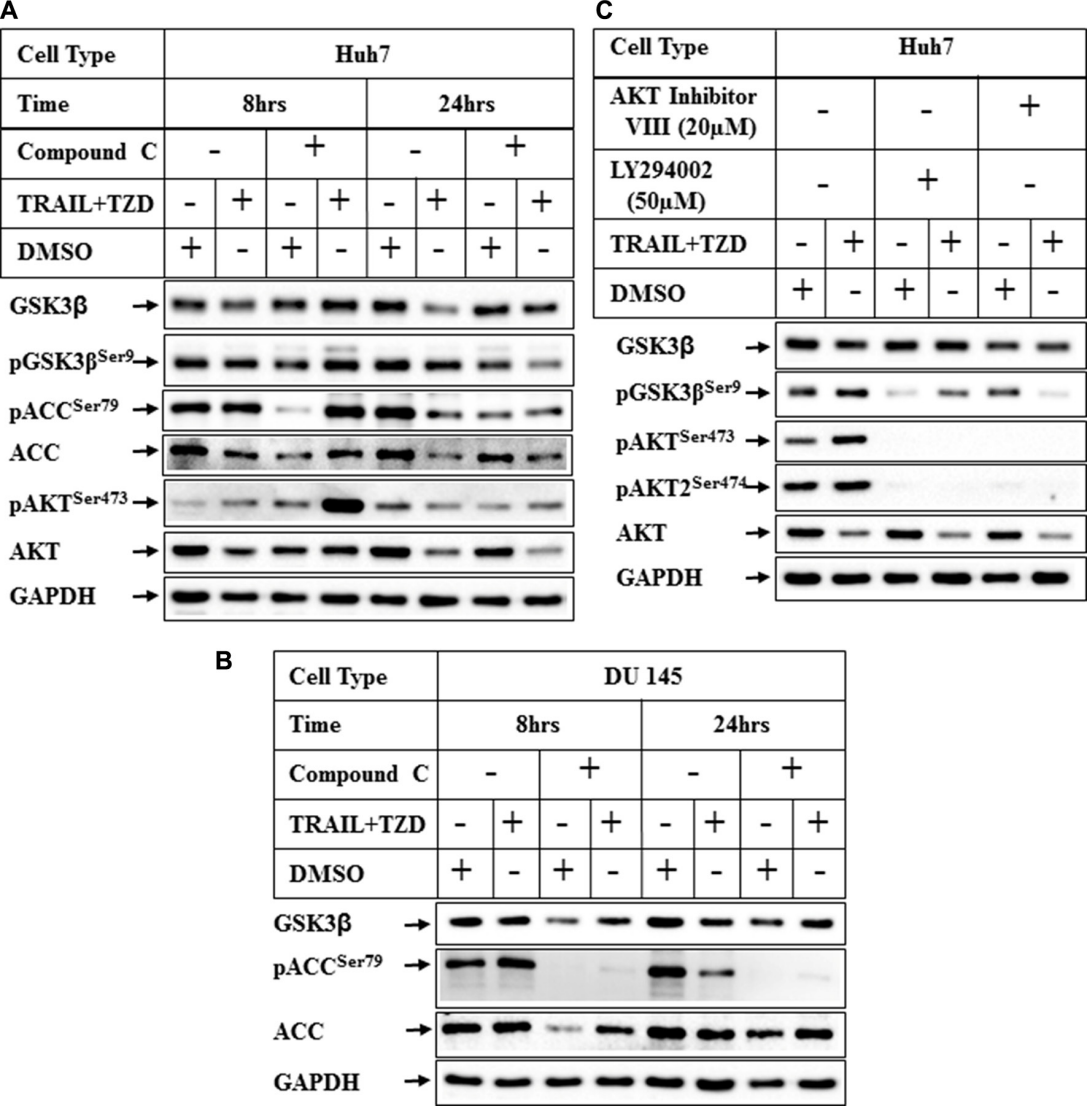
Effect of inhibition of AMPK and PI3K/AKT pathways on TRAIL-TZD-induced modulation of GSK3β pathway (**A**) Huh7 or (**B**) DU 145 cells were pretreated with 20 μM Compound C for 24 hrs followed by treatment with DMSO or TRAIL-TZD combination for 8 hrs and 24 hrs. The results were analyzed by Western blots. (**C**) Huh7 cells were pretreated with PI3K inhibitor (LY294002) or AKT Inhibitor VIII for 1 hr followed by TRAIL-TZD treatment for 8 hrs and Western blot analyses.

**Figure 7:**
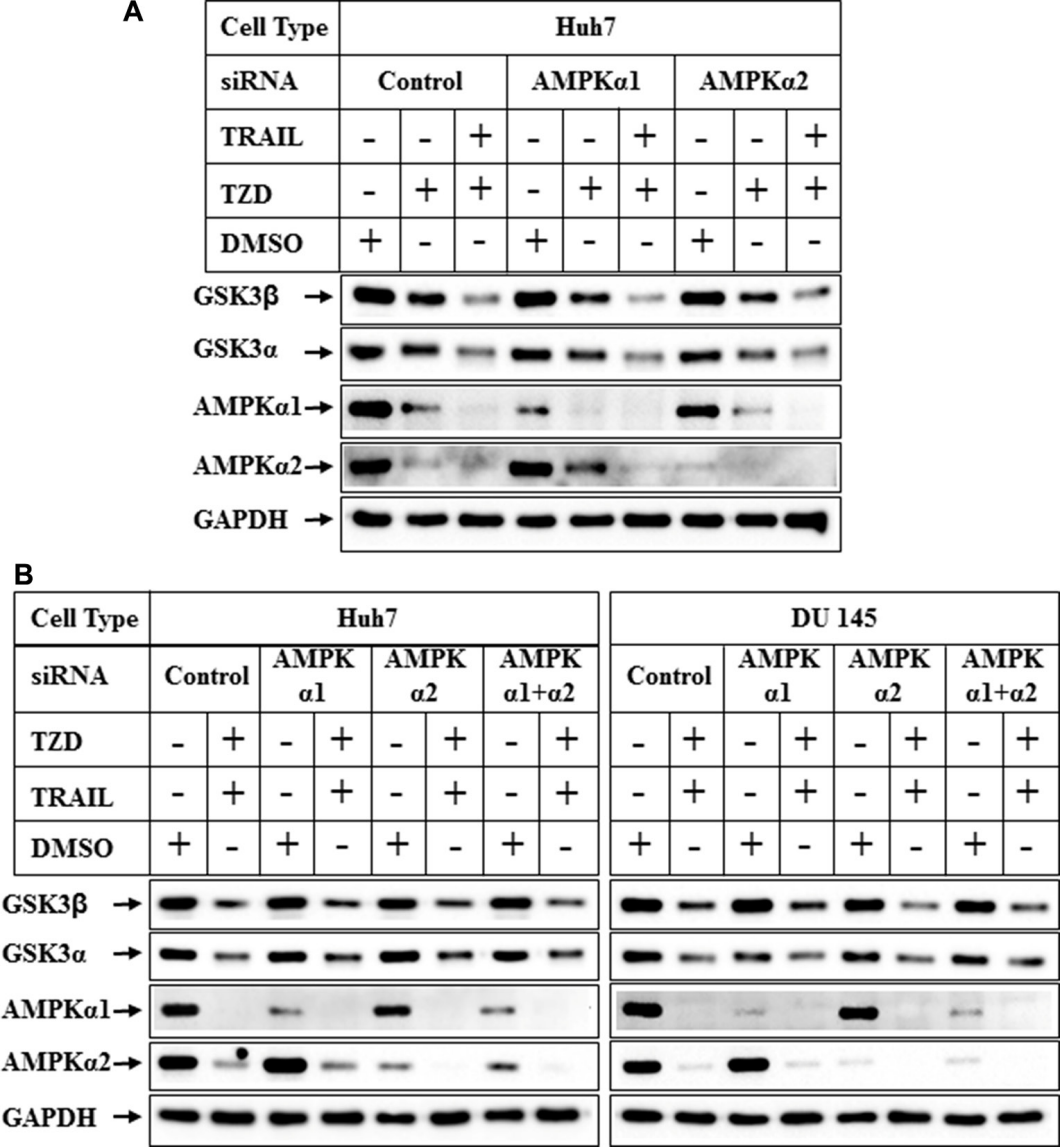
Effect of knockdown of AMPK α1 or α2 on TRAIL-TZD modulation of GSK3β pathway (**A**) Subconfluent Huh7 cells were transiently transfected with either control-siRNA, or AMPKα1-siRNA or AMPKα2-siRNA separately for 72 hrs followed by treatment with DMSO, TZD or TRAIL-TZD combination for 24 hrs. The samples were analyzed by western blots. (**B**) Huh7 and DU 145 cells were transiently transfected with either control-siRNA, AMPKα1-siRNA, AMPKα2-siRNA or a combination of AMPKα1 and AMPKα2-siRNA for 72 hrs. The cells were harvested following a treatment with DMSO or TRAIL-TZD combination for 24 hrs and analyzed by Western blots.

In recent studies we have demonstrated that TRAIL and TZD-combination can induce apoptosis in prostate cancer cells involving AMPK pathway [35]. Since TZD can also activate AMPK [36, 37], we determined any role of AMPK in attenuating GSK3β pathway by pretreating the cells with Compound C (a known inhibitor of AMPK), prior to TRAIL-TZD treatment. These results showed that TRAIL-TZD-induced attenuation of total GSK3β expression at 24 hrs can be partially antagonized by Compound C pretreatment in both Huh7 and DU145 cells (Figure 6A, 6B, compare lanes 5, 6 and 7, 8, GSK3β panel). Compound C also inhibited pACC^Ser79^ (AMPK downstream target) in both cell types (Figure 6A, 6B), indicating the efficacy of the inhibitor. This suggested a potential involvement of AMPK in mediating total GSK3β reduction. To confirm the participation of AMPK in mediating these effects, we determined the effect of TRAIL-TZD on GSK3β expression following knockdown of AMPKα. Surprisingly, siRNA-mediated knockdown of AMPKα1 or α2 alone was unable to antagonize TZD or TRAIL-TZD-induced attenuation of GSK3β expression (Figure 7A, compare lanes 2, 5 & 8 and lanes 3, 6 & 9). Knocking down AMPKα1 or α2 in combination also showed no effect in any of the cell types (Figure 7B, compare lanes 2, 4, 6 & 8). These suggested that GSK3β expression was regulated via a Compound C sensitive but AMPK-independent mechanism.

In these experiments we also observed that Compound C pretreatment was unable to antagonize TRAIL-TZD-induction of pGSK3β^Ser9^ levels and rather showed minor induction, and a corresponding hyper-activation of AKT (Figure 6A, pGSK3β^Ser9^ and pAKT^Ser473^ panels, compare lanes 2 & 4). To determine whether PI3Kinase and AKT was mediating GSK3β^Ser9^ phosphorylation in TRAIL-TZD pathway, cells were pretreated with either the PI3Kinase inhibitor (LY294002) or the AKT inhibitor (AKT inhibitor VIII) followed by TRAIL-TZD treatment. LY294002 significantly antagonized basal and TRAIL-TZD-induced GSK3β^Ser9^ phosphorylation levels which was fully antagonized by AKT inhibitor VIII (Figure 6C, compare lanes 2, 4 & 6, pGSK3β^Ser9^ panel). pAKT^Ser473^ or pAKT2^Ser474^ levels were completely abolished by LY294002, suggesting its efficacy in antagonizing PI3Kinase activation. These interesting results suggested that TRAIL-TZD likely increases GSK3β^Ser9^ phosphorylation via a mechanism that involves AKT and upstream PI3Kinase pathways.

### Effect of GSK-3β inhibition on TRAIL-TZD-induced apoptosis

**Figure 8:**
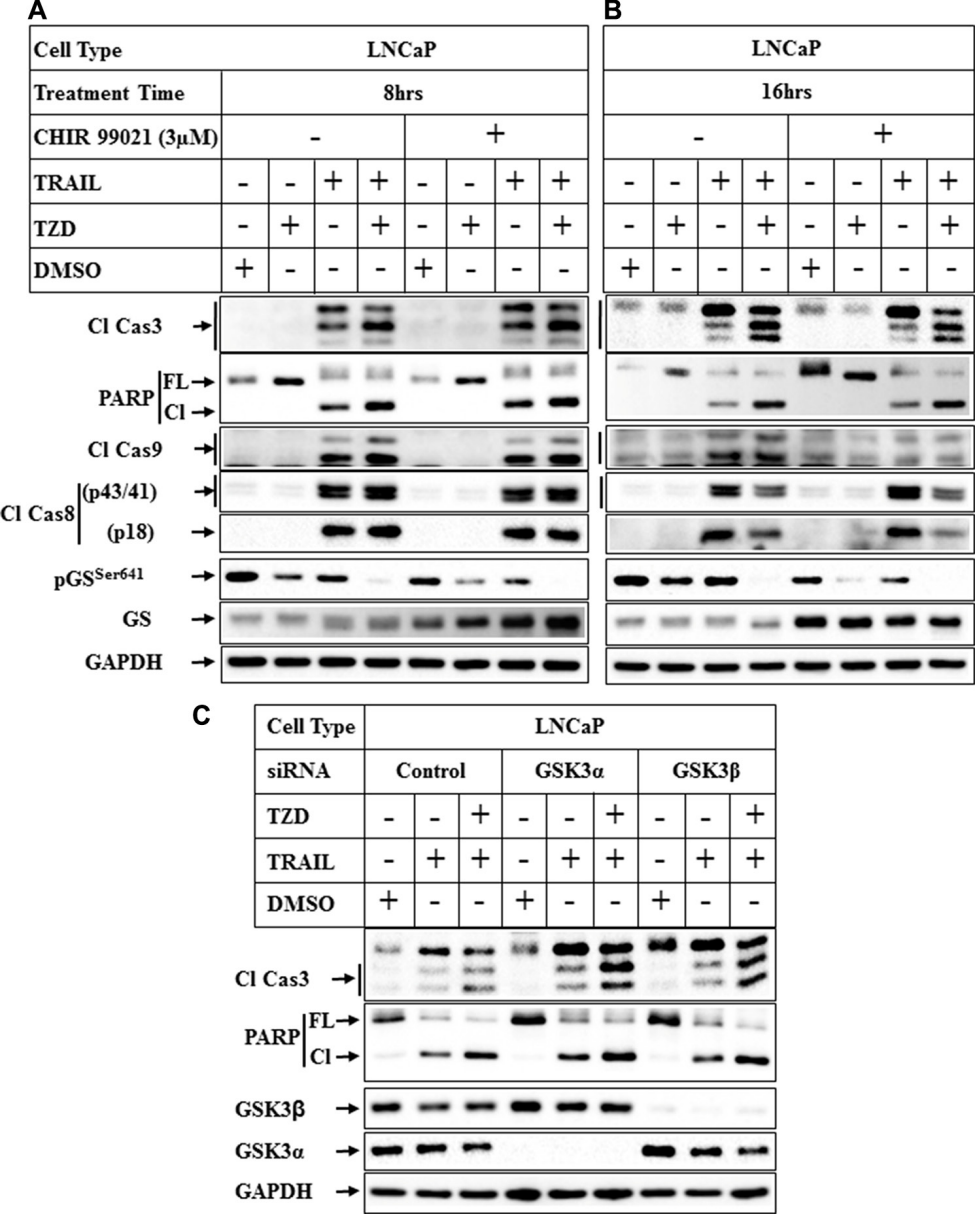
Effect of inhibition of GSK3β pathway on TRAIL-TZD-induced apoptosis LNCaP cells were pretreated with 3 μM CHIR 99021 for 1 hr followed by treatment with DMSO, TZD, TRAIL or TRAIL-TZD combination for 8 hrs (**A**) or 16 hrs (**B**). Western blot analyses were performed next with the indicated antibodies. (**C**) Subconfluent LNCaP cells transiently transfected separately with control-siRNA, GSK3α-siRNA or GSK3β-siRNA for 72 hrs followed by treatment with DMSO, TRAIL or TRAIL-TZD combination for 16 hrs. The samples were analyzed by Western blots.

To understand whether antagonism of GSK3β was necessary to promote apoptosis, cells were pretreated with pharmacological inhibitors of GSK3β followed by treatment with TRAIL or TRAIL-TZD. Pretreatment with CHIR 99021, a specific inhibitor of GSK3β [38] produced no significant increase on TRAIL or TRAIL-TZD-induced apoptosis after 8 hrs or 16 hrs of treatment (Figure 8A and 8B). CHIR 99021, however, inhibited pGS^Ser641^ levels, suggesting its efficacy in inhibiting GSK3β activity. Similar results were obtained with two other inhibitors, GSK3β inhibitor VIII and Kenpaullone (Supplementary Figure S1 and S2). To confirm the role of GSK3β in mediating apoptosis or resistance, GSK3β or GSK3α was knocked down first followed by treatment with TRAIL or TRAIL-TZD. Knockdown of GSK3α and to a lesser extent GSK3β seemed to increase TRAIL-TZD-induced Caspase 3 and PARP cleavage, (Figure 8C, compare lanes 3, 6 & 9). On the other hand, TRAIL-induced Caspase 3 and PARP cleavage was only marginally increased by GSK3α knockdown, while knockdown of GSK3β produced no major effect (compare lanes 2, 5, 8). These suggested that antagonism of both isoforms are necessary to increase TRAIL-TZD-induced apoptosis, and GSK3α seems to be important in TRAIL-induced apoptosis.

## DISCUSSION

The serine/threonine kinase GSK3β plays important roles in the pathogenesis of a wide variety of diseases that include neurodegenerative diseases (e.g. Parkinson's Disease), inflammatory diseases and cancer [39–41]. GSK3β expression is known to be induced in several cancer types, which include colon cancer [17], pancreatic cancer [18, 19], prostate cancer [20–22] and glioblastoma [23]. Increasing evidence points towards a pro-oncogenic role of GSK3β in various cancer types due to its effects in promoting cell proliferation and survival [17, 42]. Particularly, in pancreatic cancer cells GSK3β pathway was associated with increased NFκB activity, increased cancer cell survival [24], tumor dedifferentiation [19] and tumor resistance [25, 26]. There is also a strong evidence supporting a role of GSK3β in prostate cancer where it is involved in promoting androgen receptor function and nuclear translocation [20, 31, 32]. In addition, increased cytoplasmic GSK3β in prostate tumor samples correlated with the clinical stage and Gleason score [30]. While all these suggest that targeting GSK3β might be an important and effective means of controlling cancer progression, therapeutic options currently available are limited. This is because majority of the available pharmacological inhibitors of GSK3β have limited specificity and also target several other protein kinases [38].

In an attempt to overcome this limitation of GSK3β inhibitors, in the current study we aimed at elucidating the mechanism by which GSK3β expression can be antagonized in cancer cells. Although TZD-mediated antagonism of GSK3β was reported earlier [43], those studies provided limited insight towards the mechanism involved. Our results show that treatment with a combination of TRAIL and TZD which induces potent apoptosis [35], also antagonized the expression of total GSK3β in various cancer cells. More in depth analysis showed that TZD alone can significantly attenuate GSK3β expression, which inhibited total GSK3β via antagonizing its transcription. Since TZD is a ligand of PPARγ, we determined whether PPARγ played any role in this antagonism via designing siRNA-mediated knockdown experiments. Surprisingly, TZD-induced antagonism seemed to be PPARγ-independent. This is supported by several earlier studies that have showed that TZD can antagonize expression of β-catenin [44], cyclin D1 [45], c-myc [46] independent of PPARγ. Since TZD is known to activate AMPK and our earlier studies showed the involvement of AMPK pathway in TRAIL-TZD-induced apoptosis, we hypothesized that AMPK might be involved in regulating GSK3β expression. In fact, pretreatment with a pharmacological inhibitor of AMPK (Compound C) was capable of partially reversing the inhibitory effects of TRAIL-TZD on total GSK3β expression. However, these effects of Compound C seemed to be AMPK-independent, since knocking down AMPKα1 or α2 alone or in combination was unable to reverse the expression of GSK3β in the presence of TRAIL-TZD. Several studies have reported AMPK-independent effects of Compound C [47], which seems to involve antagonism of mTOR pathway in T cells [48] and multiple mechanisms in human gliomas [49]. It is unclear who the mediators are downstream of TZD or TRAIL-TZD that regulate GSK3β expression in the cancer cells. Earlier studies by Zhang et al. have shown that mutant K-Ras induces GSK3β transcription in pancreatic cancer cells via MAPK involving E-twenty six 2 (ETS2) transcription factor and p300 histone acetyltransferase [34]. Since TZD is known to inhibit the ETS pathway [50, 51], it seems possible that the inhibitory effects of TZD on GSK3β might involve ETS transcription factors. More molecular approaches are needed to firmly establish this in TZD-GSK3β axis.

To understand whether GSK3β inhibition plays a major role in mediating TRAIL-TZD-induced apoptosis or increased TRAIL sensitivity in cancer cells, we pretreated cells with different pharmacological inhibitors of GSK3β- CHIR 99021, GSK3β inhibitor VIII and Kenpaullone followed by treatment with TRAIL alone or a combination of TRAIL and TZD. These results showed that these inhibitors can successfully antagonize GSK3β pathway as indicated by the corresponding reduction of pGS^Ser641^ levels, but produced no effect on apoptosis. To validate these, we also studied the effect of knocking down either GSK3α or GSK3β on TRAIL or TRAIL-TZD-induced apoptosis. In these studies, although knocking down GSK3α and to a lesser extent GSK3β increased TRAIL-TZD-induced caspase 3 and PARP cleavage (indicating apoptosis), TRAIL-induced effects were potentiated only by GSK3α knockdown. Similar observations were also reported by other investigators, where inhibition of GSK3β in pancreatic cancer cells significantly inhibited NFκB activity but failed to sensitize to gemcitabine [18]. In a separate study, Lithium-induced inhibition of GSK3β antagonized chemotherapy-induced apoptosis [52]. On the contrary, studies in colon cancer cells showed that either pharmacological inhibition or siRNA-mediated knockdown of GSK3β augmented TZD-induced reduction of NFκB activity, cell growth inhibition and apoptosis induction [43]. The reason behind these discrepancies is not quite clear, but indicates the complexity of GSK3β pathway which might be cancer-type specific and needs further analysis. In fact, GSK3β is known to have a paradoxical role in mediating intrinsic and extrinsic pathways of cellular apoptosis [53]. Various other studies have shown the involvement of GSK3β in specific pathways of apoptosis [54, 55, 52]. As described earlier and in view of multiple cellular effects regulated by GSK3β, it is unclear at this point which major biological effects are mediated via inhibition of GSK3β. It remains a possibility that TZD-mediated antagonism of GSK3β/α axis might mediate a non-apoptotic form of cell death (or necroptosis) as was reported by others [56]. Necroptosis is a form of programmed necrosis that involves death receptors and specific signal transduction pathways mediated by receptor-interacting protein (RIP) kinases [57, 58]. TZD has also been shown to be involved in necroptosis [59]. Despite this complexity, our studies provide a novel insight towards a pathway in which TZD can antagonize GSK3β expression and might be effective in targeting those cancers which rely on GSK3β activity for proliferation and survival.

## MATERIALS AND METHODS

### Reagents and antibodies

RPMI and DMEM, DMEM F12 tissue culture media, Lipofectamine 2000 and β-galactosidase assay kit were purchased from Invitrogen; the luciferase Assay Reagent from Promega (Madison, WI); Troglitazone, TRAIL and Cycloheximide (CHX) were purchased from EMD Biosciences (Gibbstown, NJ), Compound C, AKT Inhibitor VIII, LY294002, Kenpaullone and AR-A014418 were from EMD Millipore (Billerica, MA), CHIR 99021 was from Sigma (St. Louis, MO). The antibodies utilized were obtained from the following sources: poly (ADP-ribose) polymerase (PARP), caspase-3, GSK-3β, phospho-GSK-3β^Ser9^, GSK3α, AKT, pAKT^Ser473^, pAKT2^Ser474^, PPARγ, AMPKα1 and α2, GS, pGS^Ser641^ from Cell Signaling Technologies (Danvers, MA); GAPDH from Ambion Inc. (Austin, TX). The GSK3β promoter luciferase plasmid (pGL3-GSK-3β-luc (−427/+66) was obtained from the laboratory of Dr. Daniel D. Billadeau, Mayo Clinic, Rochester, MN [34].

### Cell culture

Human Prostate cancer cells (LNCaP, DU 145), pancreatic cancer cells (BxPC3) were obtained from ATCC and maintained in RPMI (LNCaP, DU 145) and DMEM (BxPC3) media supplemented with 10% FBS, 100 IU/ml penicillin, and 100 μg/ml streptomycin. Human HCC cells (Huh7) were obtained as described [60] and grown in DMEM/F12 media. In TRAIL and TZD experiments, cells were treated with 100 ng/ml TRAIL or 50 μm TZD (unless indicated otherwise) alone or in combination for various lengths of time followed by Western blot analysis. In the studies with CHX, the cells were pretreated with 50 μg/ml of CHX for 48 hrs followed by TRAIL-TZD for 24 hrs. For the inhibitor experiments, cells were pretreated for 1 hour or 24 hours with the respective inhibitors, followed by TRAIL-TZD treatment for various lengths of time.

### Luciferase assays

Sub-confluent populations of cells were transiently transfected with GSK-3β luciferase promoter pGL3-GSK-3β-luc (−427/+66), along with β-galactosidase vector (to correct for transfection efficiency) utilizing Lipofectamine-2000 as per the manufacturer's instructions. After 24 hours of recovery in the growth medium, the cells were treated with TRAIL-TZD for various lengths of time; the cells were harvested after treatment and luciferase and β-galactosidase assays were performed as described earlier [61]. Each transfection was performed in triplicate, and each experiment was repeated at least twice. Luciferase and β-galactosidase (β-gal) assays were performed using a luminometer (Berthold Technologies, Centro XS^3^ LB 960) and a plate reader (Power Wave XS, Biotek), respectively. The results obtained were calculated as the ratio of relative light units (RLU) to β-gal values (RLU/β-gal) and expressed as the % increase compared with controls.

### Small interference RNA (siRNA)

siRNA smart pool against hPPARγ (L-003436-00), hAMPKα1 (L-005027-00), hAMPKα2 (L-005361-00), hGSK3β (L-003010-00) and hGSK3α (L-003009-00) were purchased from Dharmacon (Lafayette, CO). A negative control siRNA from Ambion Inc. (Austin, TX) was used as control siRNA. siRNA transfection was performed using Lipofectamine 2000 as per the manufacturer's instructions. Briefly, subconfluent cells plated in 35 mm plates were transfected with 50 nM of either control siRNA or target protein-siRNA for 24 h followed by recovery in serum containing medium. The transfected cells were treated after 72 hours of transfection with either DMSO or TRAIL or TZD alone or in combination for various lengths of time followed by western blot analysis.

### Western blot analysis

Western blot analysis was performed utilizing procedures described previously [60, 62]. Briefly, equal amounts of total cell extracts were fractionated by SDS-PAGE, transferred to PVDF membranes, and subjected to Western blot analysis utilizing various antibodies.

### Immunofluorescence microscopy

Immunofluorescence microscopy was performed as reported earlier [63]. Briefly, Huh7 cells plated in 4-well chamber slides were treated with DMSO, TZD, or TRAIL alone or in combination for 8 hrs and fixed with 4% paraformaldehyde in 0.1 M PBS, pH 7.4 and permeabilized with 0.1% Triton X-100 in 0.1 M PBS. Rabbit-anti GSK3β antibody and fluorochrome-conjugated goat anti-rabbit antibody was used as primary and secondary antibodies respectively. They were also stained with 4,6-diamidino-2-phenylindole dihydrochloride (DAPI) to show the nucleus. Images were captured on a Nikon ECLIPSE Ti microscope, equipped with a digital camera (Nikon DS-Qi2) at 40 × magnification.

## SUPPLEMENTARY MATERIAL FIGURES



## Abbreviations

AMPK: AMP-activated protein kinase
AR: Androgen Receptor
β-Gal: β-galactosidase
CHX: Cycloheximide
GS: Glycogen Synthase
GSK3α: Glycogen Synthase Kinase-3 alpha
GSK3β: Glycogen Synthase Kinase-3 beta
HCC: Hepatocellular carcinoma
mTOR: Mammalian Target of Rapamycin
NFκB: Nuclear Factor κB
PARP: Poly (ADP-ribose) Polymerase
PPARγ: Peroxisome Proliferator-activated Receptor gamma
RLU: Relative light units
siRNA: Small Interference RNA
TRAIL: Tumor Necrosis Factor-related Apoptosis-inducing Ligand
TZD: Troglitazone
