# Supplementary material for: Modulation of glycogen synthase kinase-3β following TRAIL combinatorial treatment in cancer cells
Source: Oncotarget. 2016 Sep 2;7(41):66892–905. doi: 10.18632/oncotarget.11834 (PMC5341845; doi:10.18632/oncotarget.11834)
Supplement: Supplementary file 1 [file oncotarget-07-66892-s001.pdf]

Modulation of glycogen synthase kinase-3β following TRAIL combinatorial treatment in cancer cells

Supplementary Materials

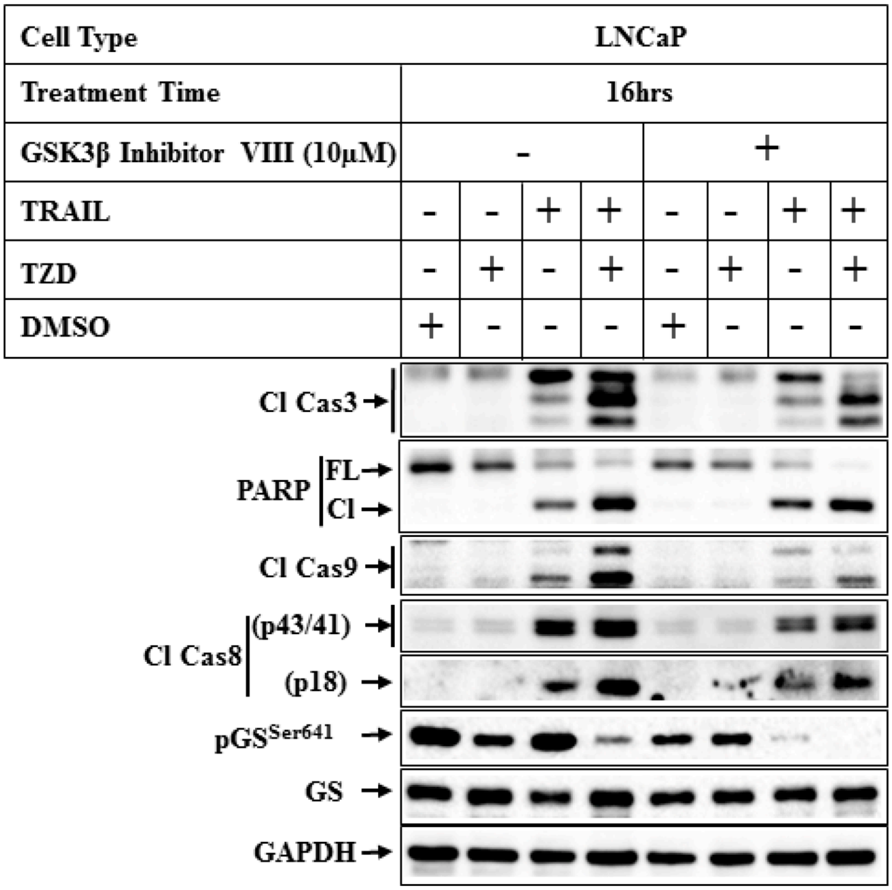

Supplementary Figure S1: Effect of GSK3β inhibitor VIII on TRAIL-TZD-induced apoptosis. LNCaP cells were pretreated with 10 μM GSK3β inhibitor VIII for 1 hr followed by treatment with DMSO, TZD, TRAIL or TRAIL-TZD combination for 16 hrs. The samples were analyzed by Western blots with the indicated antibodies.

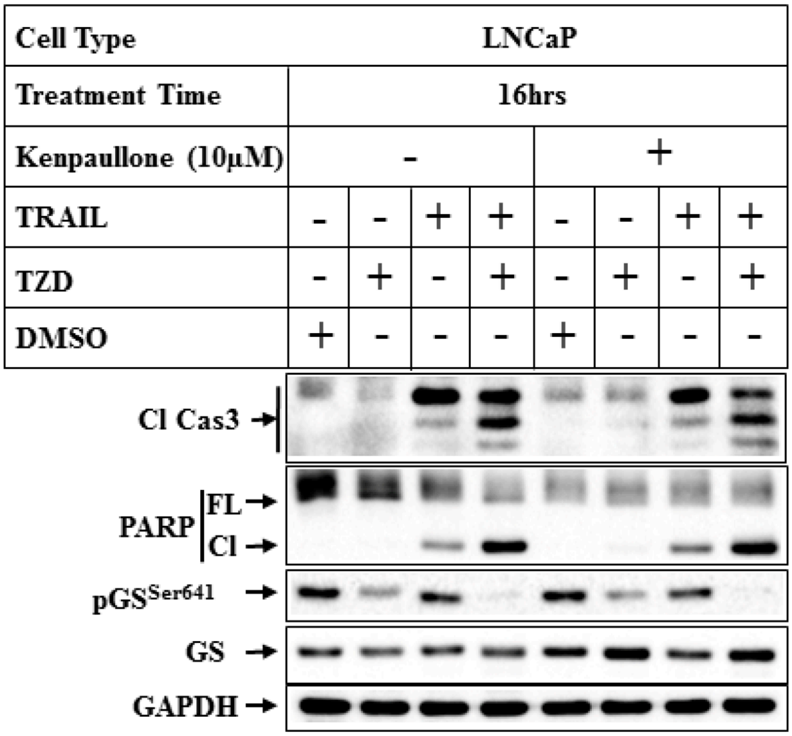

Supplementary Figure S2: Effect of Kenpaullone on TRAIL-TZD-induced apoptosis. LNCaP cells were pretreated with 10 μM Kenpaullone (GSK3β inhibitor) for 1 hr followed by treatment with DMSO, TZD, TRAIL or TRAIL-TZD combination as in Supplementary Figure S1 and Western blot analyses.
